# Berry syndrome; a successful one-stage repair in neonate periods, evaluation result after 9 years, a case report

**DOI:** 10.1016/j.amsu.2021.102200

**Published:** 2021-03-04

**Authors:** Yopie Afriandi Habibie, Pribadi Wiranda Busro, Poppy S. Roebiono, Dicky Fakhri

**Affiliations:** a Division of Thoracic Cardiac and Vascular Surgery, Department of Surgery, Faculty of Medicine, Universitas Syiah Kuala, The Dr. Zainoel Abidin General Hospital, Banda Aceh, Indonesia; b Division of Pediatric and Congenital Cardiac Surgery, Dept of Thoracic and Cardiovascular Surgery, National Cardiovascular Center Harapan Kita, Jakarta, Indonesia; c Division of Pediatric Cardiology, Dept. of Cardiology and Vascular Medicine, National Cardiovascular Center Harapan Kita, Jakarta, Indonesia

**Keywords:** Berry syndrome, Interrupted aortic arch, Aortopulmonary window, Aortic origin of the right pulmonry artery, One-stage repair

## Abstract

**Backgroud:**

IAA with an intact ventricular septum is distinctly unusual. Combination with an Aortopulmonary Window (APW), ascending aortic origin of the right pulmonary artery and PDA may be present which is called as Berry syndrome, a rare combination of cardiac anomalies, reported to be 0.046%, lethal combination and die shortly after birth.

**Case Report:**

We report a 9 days-old male neonates weighing 3.85 kg was referred by local hospital to our center and was ventilated with history of respiratory distress and severe infection since he was born. Admitted to our PCICU, 2D echo showed an IAA type A associated with a huge APW type II and restrictif PDA. A PGE1 infusion was started, during the following days the baby experienced several epileptic episodes. After improvement of the clinical condition, surgery was performed on the 20th days of life on year 2011. A successful one-stage repair of such anomalies in which cutting of PDA that arised from PA trunk and distally becoming into descending aorta, extended end to end anastomosis to conduct the ascending aortic blood flow into the descending aorta and intra arterial baffle was used. A 4-0 Gore-Tex baffle was used both to close the APW and separated the RPA from aortic origin with a good result, as his recently grown up as a cheerful 9 year old child who is growing actively and has entered elementary school in grade 2.

**Conclusion:**

Berry syndrome is a rare but well‐identified and surgically correctable anomaly. Early diagnosis and surgical treatment to avoid irreversible pulmonary hypertension is mandatory.

## Highlights

• Berry syndrome, a rare combination of cardiac anomalies, reported to be 0.046%, lethal combination and die shortly after birth. But well‐identified and surgically correctable anomaly.
• Early diagnosis and surgical treatment to avoid irreversible pulmonary hypertension is mandatory.
• Successful one-stage repair was done in a 20-days-old neonate using an alternative technique without circulatory arrest (performed on year 2011).
• Currently on year 2021, (after 9 years of evaluation) the patient has grown up as a cheerful 9 year old child who is growing actively and has entered elementary school in grade 2.
• During this patient last follow up on March 2019 at NCHHK Pediatric Cardiology clinic, echo result showed no residual AP Window, minimal pressure gradient of 18 mmHg across the aortic reconstruction with good LV function of 70%, good RV function, intact inter atrial & ventricular septum with TVG 20 mmHg. The latest echo follow up with the pediatric cardiologist showed good LV function with EF of 70%, no residual pumnonary hypertension was detected.

## 1 Background

An interrupted aortic arch (IAA) is usually associated with a ventricular septal defect and a patent duct. The coexistence of an IAA with an intact ventricular septum is distinctly unusual. In such cases, an aortopulmonary window (APW), anomalous origin of the right pulmonary artery (RPA) arising from the ascending aortais a rarely reported association of congenital heart anomalies [[1], [2], [3], [4]], first described as a syndrome by Berry only in 1992 [1]. The incidence of this combination of anomalies among patients with congenital heart disease is reported to be 0.046% [5,6] with high mortality rate (90%) in the neonatal period**,** lethal combination; most affected infants die shortly after birth [7] and the surviving patients have development of pulmonary hypertension in most cases [4,8].

Berry et al. recognized the association of the distal type of aortopulmonary window with aortic origin of the right pulmonary artery and hypoplasia or interruption of the aortic arch as a specific syndrome. Correct preoperative diagnosis and detailed anatomic depiction of each component of this association are important [7,9].

Since the first case was reported by Berry, most repairs were performed in two-stage operations, but one-stage repair has been reported recently [10]. One-stage repair in the neonatal period immediately after the correct diagnosis is mandatory. For critically ill patients, decreasing the circulatory arrest and cardiac ischemic time is important [11]. In the English literature, this anomaly has been reported in nearly 100 patients. We also experienced this rare case, and successful one-stage repair was done in a 20-days-old neonate using an alternative technique without circulatory arrest. Written informed consent from his mother has given to publish this rare case. This case report has adjusted the content to SCARE 2020 criteria [12].

## 2 Case report

A 9 days-old male neonates weighing 3.60 kg was referred by local hospital to our center (National Cardiac Center Harapan Kita Hospital/NCCHK) and was ventilated because of severe metabolic and respiratory acidosis, circulatory dynamics instability with history of respiratory distress, cyanosis, hyperbilirubinemia and severe infection that present since he was born on year 2011. The perinatal history was unremarkable and at a previous hospital she was born at 40 weeks of gestation by normal labour with a birth weight of 3.85 kg, oxygen saturation 90% and an APGAR score of 9/9. On the 6th day postpartum he presented with feeding difficulty, severe cyanosis and congestive heart failure and was referred to local hospital for further treathment, was ventilated at the emergency room with oxygen saturation 82%, and then transferred to us for possible surgical repair. Stable hemodynamic with oxygen saturation was 75%, while differential oxygen saturation was noted between the both upper and lower limbs (89%; 76% and 97%; 83%, respectively). No apparent cardiac murmur was detected. His chest X-ray showed mild cardiomegaly and congestion of the lungs, and pulmonary edema. Electrocardiogram showed normal rhythm, right axes deviation, and mild right ventricular hypertrophy.

**Fig. 1:**
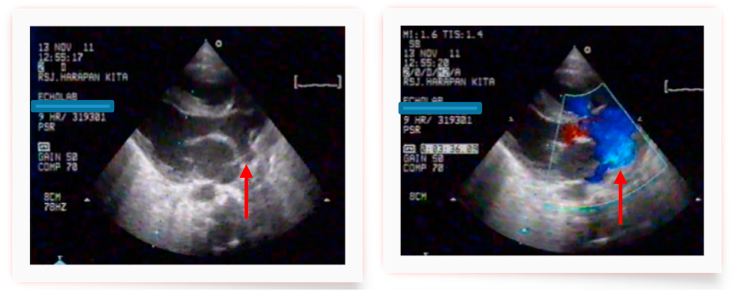
Long Axis view revealed a defect at AORPA *(Arrow)* from 2D Echocardiography and colour Doppler. (For interpretation of the references to colour in this figure legend, the reader is referred to the Web version of this article.)

**Fig. 2:**
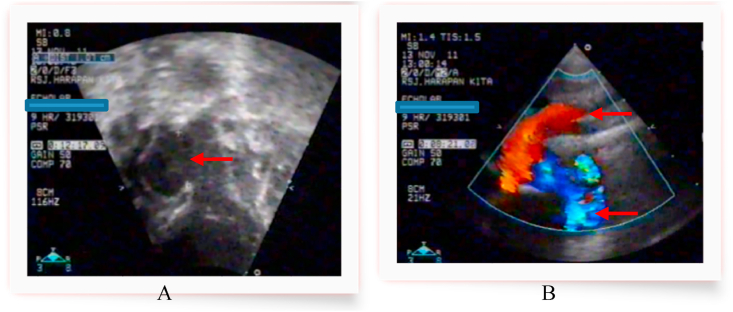
A. Apical view with tilting to the anterior view revealed a 10 mm defect of APW *(Arrow),* B. Supra sternal view showed Aortic arch with 3 branches *(upper)* and no communication with AoD. AoD arise from PDA *(lower)*.

Admitted to our pediatric cardiac intensive care unit (PCICU) with oxygen saturation between 40% and 70%. Two-dimensional Echocardiography revealed an IAA type A and APW type II with a restricted PDA, pulmonary hypertension but no ventricular septal defect (Fig. 1, Fig. 2). A PGE_1_ infusion 10 nano was started to re-open the ductus arteriosus and diuretics were administered. During the following days the baby had severe infection and DIC with increased of leucocyte 28.387, pro-calcitonine 11, D-dimer 4.800. Also had experienced of several epileptic episodes, and a brain sonography showed arachnoiditis, brain edema and no intracranial bleeding. Because of the risk of infection, sepsis and taking into account the good general condition, operation was deferred.

**Fig. 3:**
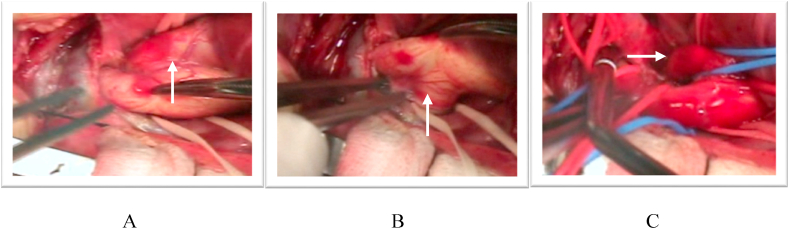
Preoperative situs demonstrating the anomalous origin of the RPA from the ascending aorta, AP Window type II and the IAA type A. PDA becoming into descending aortic **Intra Operative Findings (arrows)**:A. AP Window type II B. Ascending Aortic oringin of RPA C. Huge PDA that arise from PA Trunk and then becoming into descending aortic, confirmed the IAA type A with no communication after the 3rd arch branch.

**Fig. 4:**
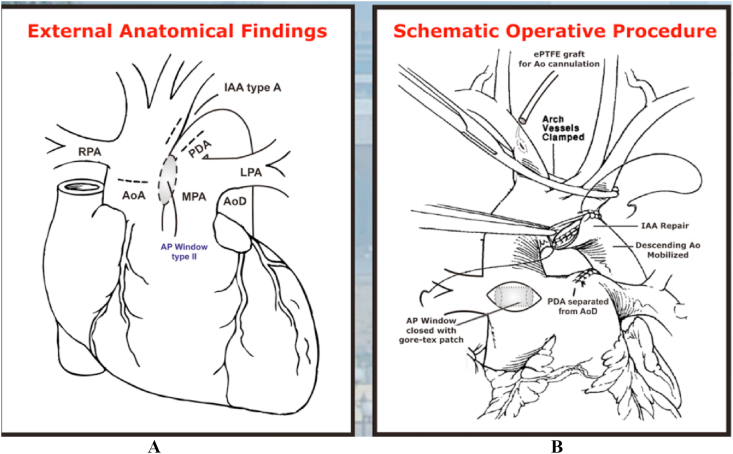
(A). Schematic drawing of intra operative findings and (B)operative surgical correction of Berry syndrome.

After improvement of the clinical condition, operation was performed on the 20th days of life. A one-stage surgical correction was performed through a median sternotomy under profound hypothermia (26 °C). Before the administration of heparin, the entire ascending aorta and the arch vessels, pulmonary trunk and both branch were mobilized. The external anatomy confirmed an IAA type A and a large APW type II, and this time the right pulmonary artery (RPA) was recognized to originate from the posterolateral part of the ascending aorta, as well as connection with common pulmonary artery. Aorta and pulmonary arteries (PA) were obvious and intact at their origin. The ascending aorta is then come to end by giving three branches including, brachiocephalic artery, left common carotid artery, and left subclavian artery. There was not evidence of descending aorta. Proximal part of PDA arose from PA trunk which is distally becoming into descending aorta with left pulmonary artery are origin from PA trunk also detected at the same time (Fig. 3, Fig. 4).

Cardiopulmonary bypass (CPB) was established by the right hemicerebral perfusion and the bicaval drainage. The right hemicerebral perfusion was obtained via expanded 4-0 mm polytetrafluoroethylene (ePTFE) graft sewn to the right brachiocephalic artery. The aortic cross clamp was applied (one-third flow) and cardioplegia was infused. Aortic arch vessels were clamped at the origin of the brachiocephalic artery, the left common carotid artery, and left subclavian artery. Immediately there after, snaring of both pulmonary arteries precluded overflooding the lungs, and the pump flow was lowered to 10% to perfuse only the myocardium. Ductal tissue was ligated near its pulmonary origin and resected until normal aortic tissue appeared, then the ascending aorta was longitudinally incised. An extended end-to-side anastomosis of the descending aorta to the distal ascending aorta was performed with a continuous 7-0 polypropylene suture. After removal of the aortic cross-clamp and off the snares in the vessels, full flow bypass was resumed and the anastomosis was checked for bleeding and undue tension.

**Fig. 5:**
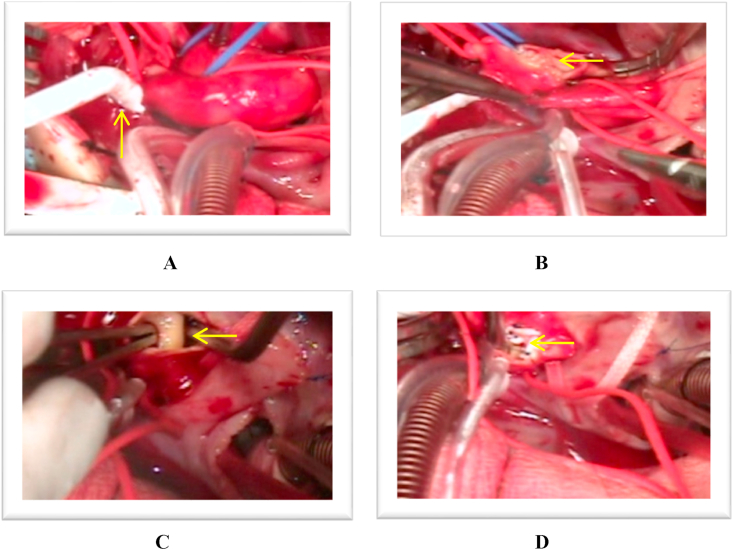
Intra operatif findings; **A***(Arrow)* shows 4-0 mm PTFE graft sewn to the right innominate artery for aortic cannulation, **B***(Arrow)* the site of the future aortic arch-descending aortic anastomosis, **C***(Arrow)* internal defect of AP window and aortic origin of RPA, **D***(Arrow)* A 4-0 Gore-tex patch was used both to close the aortopulmonary window and separated the right pulmonary artery from aortic origin.

The aortic cross-clamp was reapplied proximal to the aortic cannula, cardioplegia was infused, and the AP window repair was started. The ascending aorta was transversely incised distal to the AP window. Inspection confirmed the location of the AP window which appeared to be very large 10 mm diameter, as well as aortic origin of the RPA. The left pulmonary artery (LPA) arose from the PA trunk in the normal fashion. The coronary arteries were normally positioned. Becaused of the excessive blood flow distally, the aortic cross clamp was removed and reapplied for right hemicerebral perfusion again (one-third flow). A 4-0 Gore-tex patch was used both to close the aortopulmonary window and separated the right pulmonary artery from aortic origin (Fig. 5B).

After rewarming, CPB was easily weaned with moderate inotropic support. Total bypass time was 125 minutes, cross clamp time 71 minutes and one-third flow 66 minutes. Peritoneal dialysis was performed post operatively for 3 days and electively switch with lasix intermitten.

The postoperative recovery was uneventful. Slowly weaning from ventilation and extubated on 7th day post operatively with good clinical condition. No clinical signs of neurologic disturbances were observed and cardiopulmonary function as well as distal perfusion were very satisfactory. Early postoperative echocardiography using colour doppler demonstrated an excellent early surgical result with a minimal pressure gradient of 12 mmHg across the aortic reconstruction with good LV function, no AP window residual.

Currently on year 2021, the patient has grown up as a cheerful 9 year old child who is growing actively and has entered elementary school in grade 2. There are no abnormalities of the heart and lungs according to the patient's mother and always carries out routinely follow up with the pediatric cardiologist. However, there is still a cerebral palsy sequela which is currently being treated by a pediatric neurologist.

## 3 Discussion

The association of APW with IAA is rare and represents a life threatening malformation [13]. Only 3.5%–4.2% of patients with IAA have APW, representing 0.046% of patients with congenital heart disease [6,14]. Is currently unknown how the pathogenesis process of this anomaly occurs. However, proposed hypothesis states that the failure of formation of aortopulmonary septum leads to anomalous origin of pulmonary arteries from the undivided truncal segment [15].

IAA is characterised by a lack of luminal continuity between the ascending and descending aorta. It represents a critical ductus dependent congenital heart disease [16]. The association between IAA and APW and aortic origin of the RPA are rare, complex and represents a life-threatening malformation in the neonatal period [8,17], which poses challenging problems to both the cardiologist and the cardiac surgeon. Many forms were described according to the level of the aorto-pulmonary window and the location of the origin of the RPA. This malformation was found to be an intermediate type of truncus arteriosus, excepting that two different annulus are present [1,18,19].

Systemic circulation is ductus-dependent in this malformation, so it is very important to improve the metabolic status of these neonates before the operation with infusion of PGE_1_ and careful manipulation of artificial ventilation, if necessary, to improve systemic perfusion and prevent renal failure and low output syndrome [8]. Usually, the neonate presenting with such a complex anomaly is critically ill and may not tolerate any invasive examination; therefore a most complete diagnosis should be made by echocardiography. However, echocardiography did not allow the detection of the abnormal RPA origin in this case. Since this was recognized immediately after we opening the pericardium and did not increase the difficulty of the operation [19].

Satisfactory repair can be regarded as such only when it results in an unobstructed left ventricular outflow tract, aortic arch, RPA [[20], [21], [22]] and to restore a normal perfusion of the lower part of the body and to prevent the development of pulmonary damage [17,20]. Ideally, the growth potential of the aorta and the pulmonary artery should be maintained. The results of the one-stage repair of interrupted aortic arch and associated cardiac anomalies are relatively better than those of a two-stage approach in terms of both mortality and morbidity [11,17,19].

Surgical management has to take into account the age of the patient, the size and location of the APW, and the presence and severity of pulmonary hypertension. To correct this malformation many surgical solutions have been proposed, and there is still discussion on how close the APW (ligature, closure with a patch through the aorta or through the pulmonary artery) and how to correct the IAA [8,20]. We think that one-stage repair in the neonatal period is mandatory as soon as possible after the correct diagnosis, because the natural history of this malformation presents high early mortality and high risk of pulmonary hypertension, as previously documented in the literature [4,8,9,16].

Surgical correction of this complex anomaly requires both repair of the hypoplastic or interrupted aortic arch and septation of the APW, with exclusion of the RPA orifice from the aorta [2]. The success of this complicated repair depends on the meticulous design of each step of the procedure before surgery [9]. Most reports of the surgical correction of IAA in combination with other lesions, including AP window, have described operation on infants rather than neonates, and circulatory arrest has been used for repair. Closure of the AP window is best performed after completion of the arch repair [[20], [21], [22]].

From a technical point of view, the best surgical option is aortic arch repair by direct anastomosis, without the use of a prosthetic conduit, as described by Vouhe and associates [[22], [23]] in patients with IAA and ventricular septal defect. The APW was closed with a pericardial or Gore-Tex patch and from the pulmonary artery to avoid damage and distortion of the aortic valve. The operative risk using this technique is the same or probably less than for patients with IAA and ventricular septal defect, and the midterm results are very satisfactory [1,8,11].

In our patient, we used hypothermic bypass at low flow without circulatory arrest. Complete repair of IAA has been achieved without the use of any synthetic material, by end to end anstomosis thus allowing the potential for future growth, but for APW, and anomalous origin of the RPA we use a synthetic material (Gore-Tex) while avoiding circumferential suture lines on all the anastomoses. The posterior wall of the ascending aorta was used to form the posterior portion of the confluence between the RPA and the main pulmonary artery, and a patch 4-0 gore-tex was used to constitute its anterior half, a technique previously described by Kitagawa and colleagues [24] in a patient with a distal aortopulmonary septal defect.

**Fig. 6:**
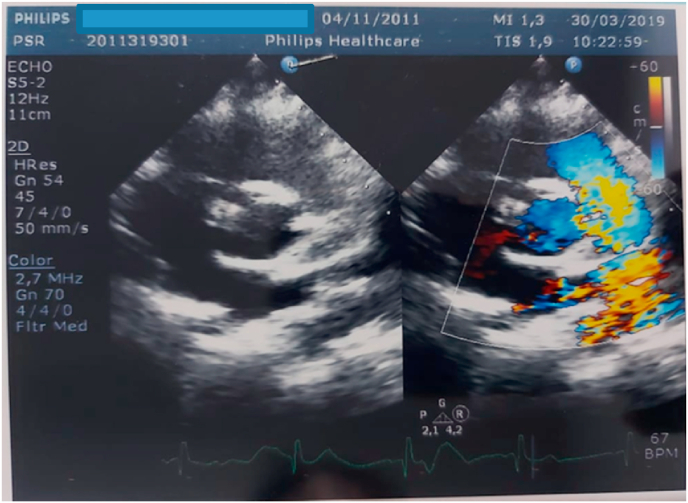
Long Axis Short axis view revealed no residual AP Window from 2D echocardiography and colour Doppler. (For interpretation of the references to colour in this figure legend, the reader is referred to the Web version of this article.)

As already recognized by other teams, the operative risk of a such complex operation may be similar to those found in patients with interrupted aortic arch and ventricular septal defect. Mid-term hemodynamic and clinical results seem to be very satisfactory [20]. During 4,5 years of follow up, an early surgical correction of APW and associated lesions showed excellent survival rate, freedom from re-intervention and no evidence of persistent pulmonary hypertension post repair [25,26]. During this pastient last follow up on March 2019 at NCHHK Pediatric Cardiology clinic, echo result showed no residual AP Window, minimal pressure gradient of 18 mmHg across the aortic reconstruction with good LV function with EF of 70%, good RV function, intact inter atrial & ventricular septum with TR Mild and TVG 20 mmHg, no residual pulmonary hypertension was detected (Fig. 6).

We believe strongly that the overwhelming majority of neonates with this anomaly are best served by a simple one-stage procedure through a median sternotomy involving direct aortic arch anastomosis and patch closure of the AP window. We hope that the long term outcome can be obtained well, as well as the process of growth and development of the patients at school age will be excellent.

## 4 Conclusion

One-stage correction of Berry syndrome in neonate is a highly risky and complex operation, but it can be safely performed in experienced centres. Echocardiography can provide adequate diagnostic information. Early surgical treatment is a therapeutic option, These patients have a good prognosis and can achieve satisfactory early and late out comes due to advances in anaesthesia, cardiopulmonary bypass, and perioperative management. In the published literature on berry syndrome there is a very high risk of death. However in our case, even though we faced all these risks, we did not experience premature or late death. Long-term results after surgical correction are excellent regardless of age or pulmonary vascular resistance.

## Declaration of competing interest

The authors declare that there is no conflict of interest regarding publication of this paper.

## Registration of research studies

1. Name of the registry: not applicable
2. Unique identifying number or registration ID: not applicable
3. Hyperlink to your specific registration (must be publicly accessible and will be checked): not applicable
